# Thymoquinone-entrapped chitosan-modified nanoparticles: formulation optimization to preclinical bioavailability assessments

**DOI:** 10.1080/10717544.2021.1927245

**Published:** 2021-05-26

**Authors:** Iqra Rahat, Syed Sarim Imam, Md. Rizwanullah, Sultan Alshehri, Mohammad Asif, Chandra Kala, Mohamad Taleuzzaman

**Affiliations:** a Department of Pharmaceutics, Glocal school of Pharmacy, Glocal University, Saharanpur, Uttar Pradesh, India; b Department of Pharmaceutics, College of Pharmacy, King Saud University, Riyadh, Saudi Arabia; c Department of Pharmaceutics, School of Pharmaceutical Education and Research, Jamia Hamdard, New Delhi, India; d Department of Pharmacognosy, Faculty of Pharmacy, Lachoo Memorial College of Science and Technology, Jodhpur, India; e Faculty of Pharmacy, Maulana Azad University, Jodhpur, Rajasthan, India

**Keywords:** Thymoquinone, nanoparticles, chitosan, polycaprolactone, pharmacokinetic

## Abstract

The major limitation with the oral administration of most of the phytochemicals is their low aqueous solubility and bioavailability. Thymoquinone (THQ) is one of the most widely used phytochemicals used to treat a variety of diseases. However, strong lipophilic characteristics limit its clinical application. Therefore, this study was aimed to design novel chitosan (C) modified polycaprolactone (PL) nanoparticles (NPs) for improved oral bioavailability of THQ. THQ-CPLNPs was optimized 33-Box–Behnken design. After that, the optimized THQ-CPLNPs was characterized by different parameters. THQ-CPLNPs showed the size, PDI, and ZP of 182.32 ± 6.46 nm, 0.179 ± 0.012, and +21.36 ± 1.22 mV, respectively. The entrapment and loading capacity were found to be 79.86 ± 4.36%, and 13.45 ± 1.38%, respectively. THQ-CPLNPs exhibited burst release in initial 2 h followed by prolonged release up to 24 h in simulated intestinal fluids. THQ-CPLNPs showed excellent mucoadhesion properties which were further confirmed with the intestinal permeation study as well as confocal microscopy. The study revealed higher permeation of THQ-CPLNPs compared to neat THQ suspension (THQ-S). Moreover, in vivo gastric irritation study revealed good compatibility of THQ-CPLNPs with the gastric mucosa. Furthermore, pharmacokinetic results depicted ∼3.53-fold improved oral bioavailability of THQ from THQ-CPLNPs than THQ-S. Therefore, from the findings, it was concluded that the prepared polymeric NPs could be an effective delivery system for improved oral bioavailability of THQ.

## 1. Introduction

From ancient times, a wide range of phytochemicals has been explored in the management of different diseases. Despite the excellent therapeutic potential of phytochemicals, several challenges still need to be resolved for the effective management of different diseases. These issues include low solubility, low gastrointestinal (GI) stability, light and heat sensitivity, systemic metabolism, and fast uptake by healthy cells/tissue. Many phytochemicals show unfavorable pharmacokinetic profiles, such as short biological half-life and fast elimination from the body (Xie et al., 2016; Rizwanullah et al., 2018).

Thymoquinone (THQ) is one of the most potent and widely investigated phytochemicals obtained from Nigella sativa. THQ is a crystalline, yellow-colored poorly water-soluble Phyto-compound with excellent therapeutic efficacy in various diseases (Elmowafy et al., 2016). As per the literature, THQ shows strong therapeutic efficacy against different ailments including gastric ulcer, diabetes, cancer, sepsis, neuroprotective, and many more (Darakhshan et al., 2015; Fakhria et al., 2019). Nevertheless, the oral bioavailability of THQ is low and requires a high dose for the management of a variety of diseases due to low stability in the GI environment. The low water solubility of THQ results in a poor dissolution in the GI fluid which in turn will result in low intestinal permeation and low oral bioavailability (Elmowafy et al., 2016; Kalam et al., 2017).

To overcome the above-discussed challenges associated with phytochemicals, research scientists across the globe worked hard and developed different nanoparticle (NP)-based delivery systems of phytochemicals for improved and targeted delivery over the past few decades. NPs enhance the stability of entrapped phytochemicals by protecting them from hostile GI environment of different pH and systemic degradation and improve their aqueous solubility and allowing controlled release and fast absorption (Ballout et al., 2018; Rathore et al., 2020).

Polycaprolactone (PL), a US-FDA approved, biodegradable and biocompatible synthetic polymer, is a widely used biomaterial for the delivery of a variety of drugs to achieve excellent therapeutic potential. PL-based NPs offer the ability of sustained drug release, protect the encapsulated drugs from different physiological barriers, and reduced dose-related side effects (Guarino et al., 2017; Shahab et al., 2020). PL produces spherical NPs and a negative charge on the surface of the NP, which provides stability to the NPs from the hostile pH of the biological system (Manjili et al., 2018). However, oral delivery of drugs through PL NPs is still challenging due to the lack of mucoadhesive property to the GI mucosa that also has a negative charge. Therefore, the coating of NPs can be done to impart a positive charge on their surface to improve mucoadhesion thereby absorption of encapsulated drugs.

Chitosan is a natural cationic copolymer of glucosamine and N-acetyl-glucosamine, is an extensively used biomaterial for oral delivery of several phytochemicals. Unique properties such as excellent biocompatibility and biodegradability, nontoxicity, stability in hostile GI media, excellent mucoadhesive, and intestinal permeability enhancing characteristics make a convenient drug delivery vehicle for a variety of drugs (Mohammed et al., 2017; Wong et al., 2018). Surface modification with CS provides a positive charge to the NP surface, which increases the adhesion of NPs on the intestinal mucosa which in turn provides significantly higher residence time to absorb the drug from the intestine (Aldawsari et al., 2020; Gilani et al., 2021). Therefore, modification of PL NPs with chitosan can significantly increase the oral bioavailability of phytochemicals, reduce dosing frequency as well as adverse effects.

Therefore, this work aimed to develop THQ encapsulated chitosan modified PL nanoparticles (THQ-CPLNPs) for improved oral bioavailability. The NPs were optimized by the Box–Behnken design and the optimized THQ-CPLNPs was further evaluated for THQ release, mucoadhesion, intestinal permeation and confocal microscopy. Finally, in vivo absorption study was assessed to evaluate the ability of THQ-CPLNPs for the improvement of oral bioavailability and the results were compared with THQ suspension (THQ-S).

## 2. Materials and methods

### 2.1. Materials

Thymoquinone (THQ), Chitosan (C; MW: 100–300 kDa; 85% deacetylated; viscosity 20 cp), polycaprolactone (PCL; average MW: 10 kDa), polyvinyl alcohol (PVA; MW: 31–50 kDa; 80–90% hydrolyzed), and type II porcine stomach mucin were purchased from Sigma-Aldrich, Saint Louis, USA. Sterile membrane filters with 0.22 and 0.45 μm pore were taken from Merck Millipore (Darmstadt, Germany). Milli Q water and AR grade chemicals were used in the experiments. The permeation and pharmacokinetic study were performed on the Wistar albino rats of either sex. The animals were housed under standard storage conditions and fed with pellet diet (Lipton, Mumbai, India). The study design was approved by Bilwal Medchem and Research Laboratory Pvt. Ltd, Jaipur Rajasthan with animal ethical committee (Reg. No:- 2005/PO/RcBT/S/18CPCSEA, Approval number:- BMRL/2021-08).

### 2.4. Experimental design

The different formulation variables were statistically optimized by BBD design. The prepared NPs THQ-CPLNPs optimized using the Design Expert® software (V-12.0 Stat-Ease Inc., Minneapolis). The independent factors were: PCL (A; 35–65 mg), CS (B; 0.3–07%), and PVA (C; 2–3%). The low, medium and high levels of the factors were selected as (−1, 0, and +1), and their representative actual values are demonstrated in Table 1. The dependent factors were selected as PS (*Y_1_*), PDI (*Y_2_*), and %EE (*Y_3_*). As per the design, the prepared 15 formulae with 3 center points is represented in Table 2. The optimized THQ-CPLNPs was selected based on low PS, PDI, and high %EE as per the point prediction method. The influence of independent factors on all the responses and interaction between factors was assessed by statistical analysis. Analysis of variance (ANOVA) was performed for model estimation and term significance. Probability *p*-values (*p* < .05) represents the significance of the model.

**Table 1.** Various independent and dependent variables used in the Box–Behnken design for the preparation of thymoquinone encapsulated chitosan modified polycaprolactone nanoparticles (THQ-CPLNPs).

Factor	Levels used, actual (coded factor)		
Independent variables	Low (‒1)	Medium (0)	High (+1)
X_1_ = Polycaprolactone (mg; w/v)	35	50	65
X_2_ = Chitosan (%; w/v)	0.3	0.5	0.7
X_3_ = PVA (%; w/v)	2	2.5	3

Dependent variables	Goal
*Y*_1_ = Particle size (PS; nm)	Minimize
*Y*_2_ = Polydispersity index (PDI)	Minimize
*Y*_3_ = Entrapment efficiency (EE; %)	Maximize

**Table 2.** Observed Box–Behnken experimental runs of thymoquinone encapsulated chitosan modified polycaprolactone nanoparticles (THQ-CPLNPs) with their experimental value.

Runs	(A) Polycaprolactone	(B) Chitosan	(C) Polyvinyl alcohol	*Y*_1_ Particle size	*Y*_2_ (Polydispersity index)	*Y*_3_ (Entrapment efficiency)
	mg	%	%	nm		%
F1	35	0.7	2.5	164.12	0.16	76.46
F2	35	0.5	2	159.08	0.19	72.08
F3	50	0.7	2	211.07	0.19	81.49
F4	65	0.5	2	225.14	0.23	87.78
F5	50	0.3	3	176.83	0.16	78.49
F6	65	0.5	3	203.97	0.25	90.54
F7	50	0.7	3	186.28	0.19	87.18
F8	50	0.5	2.5	182.48	0.17	80.59
F9	50	0.5	2.5	184.96	0.18	79.72
F10	65	0.7	2.5	227.15	0.23	92.37
F11	65	0.3	2.5	210.06	0.21	86.89
F12	35	0.5	3	146.51	0.14	74.12
F13	50	0.5	2.5	183.54	0.19	78.67
F14	50	0.3	2	189.02	0.21	78.71
F15	35	0.3	2.5	144.56	0.14	70.47

### 2.5. Formulation of NPs

THQ-CPLNPs were prepared as per the reported method single emulsion solvent evaporation method with slight modification (Lima et al., 2018; Alshehri et al., 2020). Firstly, two separate phases, i.e. organic and aqueous phase was prepared. The organic phase was prepared by adding the weighed quantity of THQ (20 mg) and PCL (35 − 65 mg) in 1 mL dichloromethane. An aqueous phase was prepared dissolving CS (0.3−0.7%, w/v) in 1% v/v acetic acid solution. After that PVA (35%) is dissolved in aqueous phase. Then, organic phase was added dropwise to the aqueous phase with continuous stirring at 800 r.p.m. At last, the obtained dispersion was sonicated (Hielscher, Ultrasound UP-50H, Teltow, Germany) for 3 min to get nanosized THQ-CPLNPs. The organic phase was evaporated in a rotary evaporator under vaccum condition. The NPs were collected after the ultracentrifugation and stored for furthere use.

### 2.6. Characterization

#### 2.6.1. Particle evaluation

The prepared polymeric NPs were characterized for different parameters like particle size (PS), polydispersibility index PDI, zeta potential (ZP). The prepared NPs (0.1 mL) were taken and further diluted 100 fold to assess the parameters using a zeta sizer (Malvern Instruments Ltd., Worcestershire, UK). The samples were diluted to avoid the multi scattering of the particle in tested samples. The ideal PDI value must be less than 0.5, and the ZP value must be between ± 10–30.

#### 2.6.2. Encapsulation and loading efficiency

The drug encapsulation (EE) and loading (DL) efficiency of the prepared THQ-CPLNPs were evaluated by the centrifugation method (Abd El Hady et al., 2019). The samples (5 mL) were taken and centrifuged at 15,000 rpm for 30 min using a cooling centrifuge (C24, REMI, Mumbai, India). The supernatant was collected, diluted and THQ content in each NPs were quantified using UV spectrophotometer (Shimadzu 1700, Shimadzu Corp., Kyoto, Japan). EE and DL were calculated using the below following equations

$$\%\mathrm{EE}=\frac{\text{Total TQ}-\text{TQ in supernatant}}{\text{Total TQ}}\times 100$$

$$\%\mathrm{DL}=\frac{\text{Total TQ}-\text{TQ in supernatant}}{\text{Weight of NPs}}\times 100$$

#### 2.6.3. Drug release

THQ release from optimized THQ-CPLNPs and THQ-S was performed by the diffusion technique using a dialysis bag (Badran et al., 2018). The experiment was performed by using 500 mL of SGF (pH 1.2) and SIF (pH 6.8) as dissolution media. The sample containing THQ (∼5 mg) was filled in the dialysis membrane (Sigma-Aldrich, St. Louis, MO) and dipped into dissolution media. The release media was stirred at a speed of 100 rpm and the at predetermined time points. 2 mL released THQ content was withdrawn and replenished with the same media to make uniform volume throughout the study. A similar procedure was performed with THQ-S. The released THQ content was filtered, diluted, and quantified using a UV spectrophotometer. The released content was further evaluated for the release mechanism of THQ from the prepared NPs. The data fitted into different release kinetic models like Zero order, First order, Higuchi model, and Korsmeyer Peppas model (Ritger & Peppas, 1987). The selection of the best fit model was done based on the model that showed the highest regression coefficient.

#### 2.6.4. Mucoadhesion study

The mucoadhesion experiment was assessed to evaluate the adhesive properties of THQ-CPLNPs to the mucous membrane as per the reported procedure with slight modification (Pauluk et al., 2019; Coutinho et al., 2020). Briefly, the mucin solution was mixed with THQ-CPLNPs (1:1 ratio) and incubated at 37 °C with continuous stirring for 3 h. The incubated mixture was centrifuged at 14,000 rpm for 30 min at 4 °C, supernatant was removed, diluted appropriately and free mucin content was evaluated by UV-Vis spectrophotometer at 258 nm. The same experimental procedure was followed for THQ-S. The mucoadhesive efficiency was calculated by the following equation:

$$\text{Mucoadhesive efficiency }(\%)=\frac{\text{Initial mucin content}-\text{Free mucin content}}{\text{Initial mucin content}}\times 100$$

#### 2.6.5. Permeation study

The comparative permeation study of THQ-CPLNPs and THQ-S was performed as per the previously reported procedure (Jha et al., 2014). The rats fasted overnight with free access to water. The rats were sacrificed, the intestine was removed and washed three times with water to remove the food content. The intestine was cut (5 cm long) and filled with THQ-CPLNPs and THQ-S (∼5 mg of THQ). The study was performed in Kreb’s solution (250 mL) with a continuous flow of 95% oxygen using an aerator. The sample-filled intestines were dipped in Kreb’s solution for the specified time and at each time point, the permeated THQ content (2 mL) was removed and replaced with fresh Kreb´s solution to maintaining the uniform condition throughout the study. The released content was filtered, diluted and THQ content at each time point was evaluated by HPLC method (Gilani et al., 2019). From the result, further permeation flux and apparent permeability coefficient (APC; $P_{\mathrm{app}}$) were calculated using the below equation (Alhakamy et al., 2020):

$$\mathrm{APC}=\frac{\mathrm{flux}}{\text{Surface area}\times \text{Loaded THQ content}}cm/\min$$

#### 2.6.6. Permeation depth

The permeation depth of the prepared THQ-CPLNPs was evaluated by confocal laser scanning microscopy (CLSM; Olympus FluoView^TM^, Hamburg, Germany) (Dayan & Touitou, 2000). The procedure followed for this study was the same as the permeation study. The test sample containing 0.03% Rhodamine B loaded CPLNPs was filled in the intestinal sac and tightly ligated. The sac was kept in a beaker containing Kreb’s solution (250 mL) and oxygenated with 95% oxygen using an aerator. The study was performed at 37 °C with a stirring speed of 50 rpm for 3 h. The excess dye from the membrane was removed with washing three times. The membrane was cut longitudinally and fixed on the glass slide, and the permeation depth was analyzed using a microscope at 514 nm fluorescence excitation. A similar procedure was performed for the neat 0.03% Rhodamine B solution for the comparison.

#### 2.6.7. Gastric mucosa irritation study

The comparative gastric irritation study between THQ-CPLNPs vis a vis THQ-S was assessed on the Wistar albino rats as per the reported procedure (Sharma et al., 2019). The rats were distributed into three groups as Group I treated with normal saline, Group II treated with THQ-S, and Group III treated with THQ-CPLNPs. The animals were sacrificed after 2 h of treatment using anesthesia, and the stomach was removed and rinsed with Kreb’s solution to remove the food residue. The treated specimen was stored in formaldehyde (10%, v/v) and the gastric tissue was sliced and blocks were prepared using paraformaldehyde (4%). The section was cut and further stained using hematoxylin and eosin (H&E) to check the internal damage under the electron microscope (MOTIC, Tokyo, Japan).

#### 2.6.8. Pharmacokinetic study

Pharmacokinetics of THQ-CPLNPs and THQ-S was examined in Wistar albino rats (220-250 g) as per the reported procedure with slight modification (Kalam et al., 2017). Before the experiment, animals were fasted overnight and randomly grouped as THQ-CPLNPs-treated group and THQ-S treated group with six animals in each group. THQ-S was developed by gentle mixing of THQ with sodium carboxymethyl cellulose (0.5% w/v) in double-distilled water. The animals were administered with THQ-CPLNPs and THQ-S (dose 20 mg/kg). After oral administration, 500 μL of blood was collected via the ocular vein in a heparinized tube at 0.5, 1, 2, 4, 6, 8, 12, and 24 h. The blood samples were centrifuged at 10,000 rpm for 5 min to separate the plasma and stored for −20 °C. After that, the deproteinization procedure of plasma samples was carried out by adding 1 mL acetonitrile in each sample. Plasma samples were then vortexed for 10 min and further centrifuged for 10 min at 6000 rpm. The supernatant was collected and THQ content in plasma at predetermined time intervals is quantified by RP-HPLC. Finally, different pharmacokinetic parameters, like peak plasma concentration (*C*_max_), time to reach peak concentration (*T*_max_), mean residence time (MRT), area under the curve (AUC) and elimination rate constant (*K*_el_), plasma half-life (*t*_1/2_) of THQ was calculated using the PK Solver 2.0 software by non-compartmental analysis.

#### 2.6.9. Statistical analysis

The experimental results were calculated in triplicate and presented as mean ± SD. The data were statistically evaluated by Student’s *t*-test and ANOVA using GraphPad Prism (InStat 7; San Diego, USA) at a significance level of *p* < .05.

## 3. Results and discussion

### 3.1. Statistical optimization

**Figure 1.**
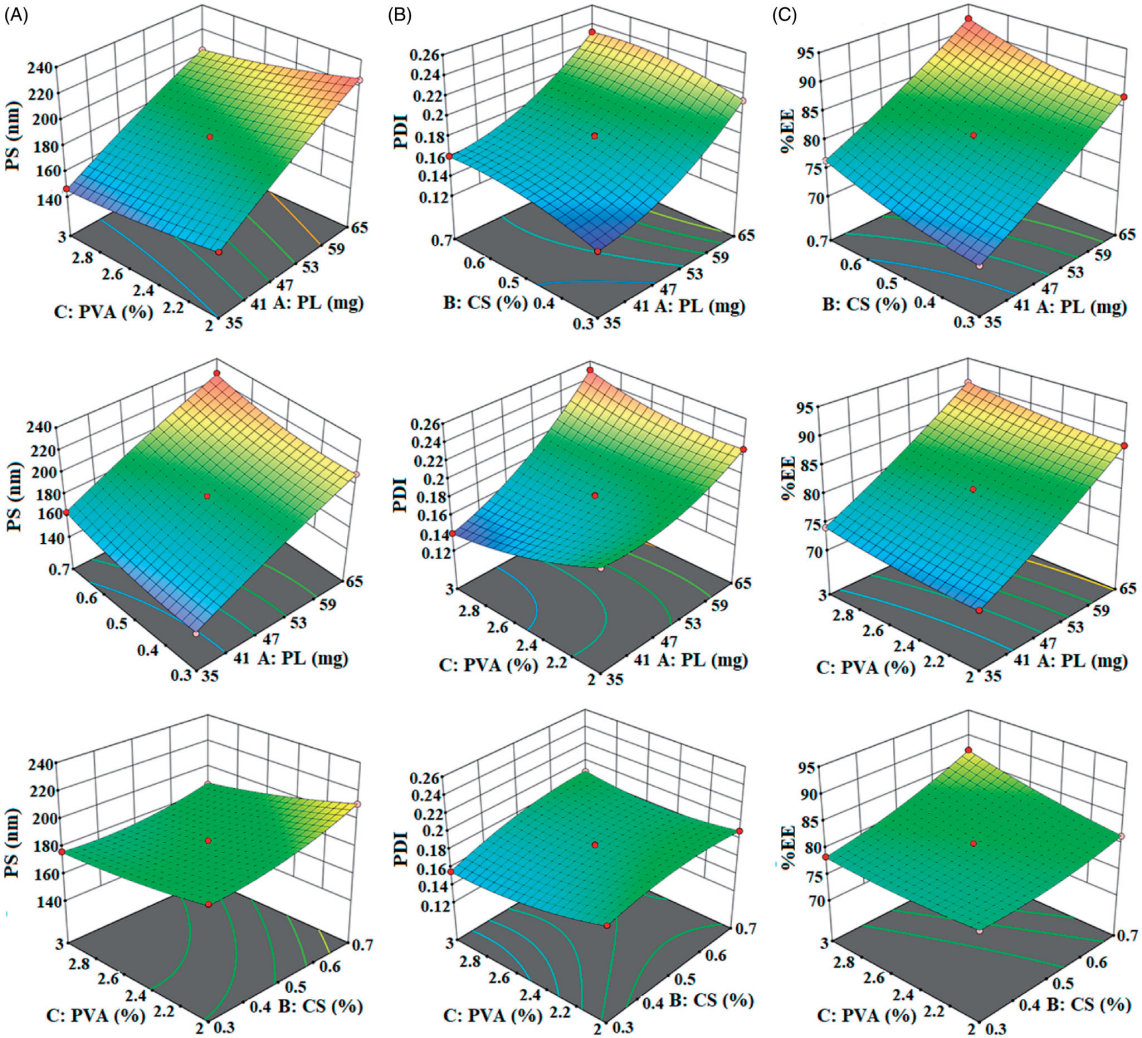
Effect of independent variables polycaprolactone, chitosan and polyvinyl alcohol on (A). size, (B). PDI, and (C). encapsulation efficiency.

The NPs were prepared by the method and optimized using three-factor at three-level. The design showed fifteen formulation runs with three center point to check the formulation error. The responses PS (*Y*_1_), PDI (*Y*_2_), and %EE (*Y*_3_) were fitted to different kinetic model to interpret the results (Table 3). The linear regression equation was used to evaluate the result by evaluating the quadratic model, linear model and two-factor interaction model. The actual and predicted R^2^ value for each response was found closer to each other. Each independent variable used in the study showed individual effect as well as the combined effect on the PS, PDI, and EE. Each response was further evaluated for the analysis of variance, and the results showed the value *p* < .0001 (Table 4). The used models were considered statistically with the *p*-value must be <.05. The results were evaluated with a polynomial equation (Equations (1)–(3)) and response surface plot (Figure 1) for each factor. The positive sign of the polynomial equal gives the synergistic effect and the negative sign depicts the antagonistic effect of each independent variable. The below polynomial equation for each response was shown by the design expert software:

(1) $$\text{Particle size}=+183.66+31.51A+8.52B‒8.84C‒0.6175AB‒2.15AC‒3.15BC‒2.16A^{2}+4.97B^{2}+2.17C^{2}$$

(2) $$\text{Polydispersity index}=+0.1793+0.0367A+0.0098B‒0.0072C‒0.001AB+0.018AC+0.0065BC+0.0148A^{2}‒0.0077B^{2}+0.0078C^{2}$$

(3) $$\text{Entrapment efficiency}=+79.99+8.06A+2.87B+1.28C‒0.1275AB+0.18AC+1.48BC+0.6083A^{2}+0.9458B^{2}+0.5283C^{2}$$

**Table 3.** Summary of regression analysis for particle size (PS; *Y*_1_), polydispersity index (PDI; *Y*_2_), and entrapment efficiency (EE; *Y*_3_) for fitting data to different models.

Model	*R* ^2^	Adjusted *R*^2^	Predicted R^2^	SD	Desirability	Remark
Response (*Y*_1_)					0.969	
Linear	0.9786	0.9727	0.9567	4.27		‒
2F1	0.9850	0.9737	0.9308	4.19		‒
Quadratic	0.9988	0.9967	0.9860	1.47		Suggested
Response (*Y*_2_)					0.985	
Linear	0.8117	0.7604	0.6103	0.0159		‒
2F1	0.9112	0.8446	0.5840	0.0128		‒
Quadratic	0.9993	0.9981	0.9971	0.0014		Suggested
Response (*Y*_3_)					0.977	
Linear	0.9763	0.9698	0.9560	1.15		‒
2F1	0.9908	0.9840	0.9801	0.8378		‒
Quadratic	0.9990	0.9973	0.9964	0.3455		Suggested

**Table 4.** Analysis of variance of response surface quadratic model of each response.

Model	Source	PS	PDI	%EE
Regression analysis				
Quadratic	Sum of squares	9336.16	0.0148	612.14
	df	9	9	9
	Mean square	1037.35	0.0016	68.02
	*F*-Value	477.52	806.29	569.74
	*P*-value, Prob > F	<0.0001	<0.0001	<0.0001
	Remark	Suggested, significant		
Lack of fit tests				
Quadratic	Sum of squares	7.77	1.500E-06	0.0616
	df	3	3	3
	Mean square	2.59	5.000E-07	0.0205
	*F*-Value	1.67	0.1154	0.0768
	*P*-value, Prob > F	0.39	0.94	0.96
	Remark	Suggested, not significant		

#### 3.1.1. Influence of the investigated factors (PL, chitosan, PVA) on PS

The prepared THQ-CPLNPs should be small enough to enhance penetration and membrane adhesion and to control the drug release for desirable therapeutic response (Delan et al., 2020). As shown in Table 2, the PS of THQ-CPLNPs ranged from 144.56 nm (F15) to 227.15 nm (F10), suggesting the capacity to produce small NPs. ANOVA analysis revealed that all the independent variables, i.e. PL (A) and chitosan (B), and PVA (C) were the significant factors affecting PS (*p* < .0001). As shown by the 3D surface plot illustrated in Figure 1(A), an increase in PCL and chitosan the PS of the NPs significantly increases. Higher polymer concentration increases the viscosity of the formulation, which leads to the enhancement of PS (Lepeltier et al., 2014). Also, high viscosity decreases the diffusion of the drug from NPs, which is another factor for enhanced PS (Tavares et al., 2017). The variable PVA (C) showed a strong negative impact on the PS. Increment in the concentration of PVA from 1.5 to 2.5% significantly (*p* < .05) decreased the PS. PVA is a surfactant and stabilizer that helps in the emulsification of polymer in the aqueous phase and prevents aggregation of NPs (Almeida et al., 2019).

#### 3.1.2. Influence of the investigated factors (PL, chitosan, PVA) on PDI

PDI value assures the homogeneity and size distribution of NPs. Generally, PDI < 0.4 is an accepted value. The PDI of THQ-CPLNPs varied from 0.14 (F12) to 0.25 (F6), as shown in Table 2. The result suggested an acceptable PS distribution and a reproducible method of preparation (Bihari et al., 2008). ANOVA analysis revealed that all the independent variables i.e. PCL (A) and CS (B), and PVA (C), significantly affected the PDI of the THQ-CPLNPs. As shown by the 3D surface plot illustrated in Figure 1(B), an increase in PL and CS, the PDI of the NPs significantly increases. An increment in PL concentration leads to the agglomeration of NPs that results in the development of NPs of different sizes (Snehalatha et al., 2008). Furthermore, the electrostatic repulsion between chitosan molecules, as a result of interchain and intramolecular hydrogen bonding, increased by increasing the chitosan concentration above an equilibrium point. This could lead to rearrangement and aggregation of chitosan molecules, thereby forming NPs of different sizes (Bihari et al., 2008). Whereas, PVA (C) showed a strong negative impact on the PDI. The surfactants decreased the interfacial tension between the aqueous and organic phases and led to the development of uniform primary emulsion during the development of THQ-CPLNPs (Sharma et al., 2016).

#### 3.1.3. Influence of the investigated factors (PL, chitosan, PVA) on %EE

EE of THQ-CPLNPs ranged from 70.5 (F15) to 92.37% (F10), as shown in Table 2, indicating that THQ was highly entrapped by the NPs. There was a significant difference in the entrapment of THQ observed in the CPLNPs. The variation in the entrapment due to a change in the composition of CPLNPs. The low, medium, and high level of each variable affect the entrapment of lipophilic THQ. The polynomial Equation (3) showed that the variables PL (A), chitosan (B), and PVA (C) given a positive effect on encapsulation efficiency. The effect of each variable was further evaluated by the 3D surface plot (Figure 1(C)). The surface plot also showed a positive effect on THQ encapsulation. As the concentration of PL (A), CS (B), and PVA (C) increases the higher concentration of THQ entrapped in the available polymer. There was enhanced drug miscibility in the organic solvent was achieved which helps to influence the THQ entrapment. The drug has shown greater solubility in the used polymer composition due to greater emulsification property in the presence of PVA (Delan et al., 2020). The surfactant and stabilizer also enhance the solubility of insoluble THQ. It helps to enhance the entrapment of THQ in available polymer PL (A) and chitosan (B). The third variable PVA (C) showed the enhanced solubility of THQ due to greater emulsification property and lead to achieve high solubility as well as an increase in EE (Kim et al., 2008; Anwer et al., 2019).

#### 3.1.4. Optimized composition

**Figure 2.**
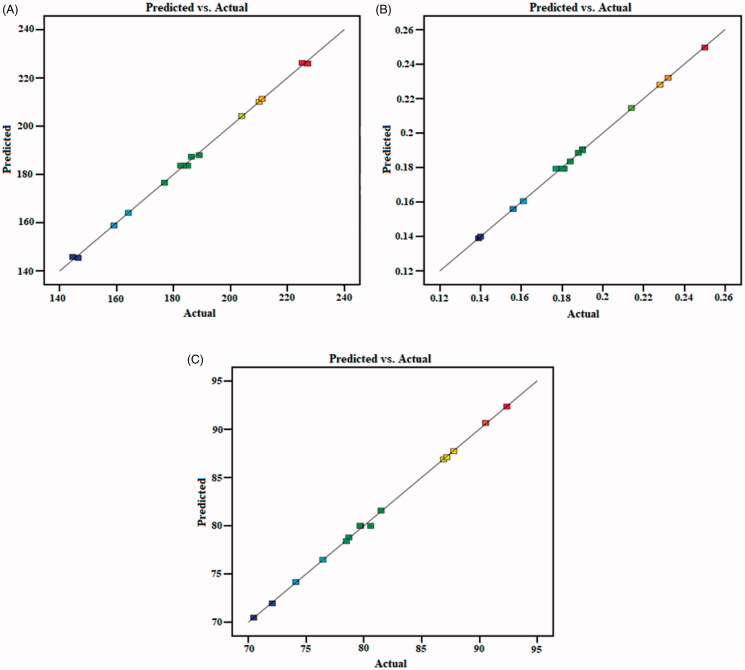
Predicted and actual value graph of the (A). particle size; (B). PDI; (C). encapsulation efficiency.

The optimized THQ‐CPLNPs was selected on the criteria of small PS, PDI, and high %EE among 15 compositions, upon ‘trading off’ different responses (*Y*_1_, *Y*_2_, and *Y*_3_) using the numerical desirability function as shown in Table 3. The ideal desirability range exists between 0 and 1. A value closer to zero means the method is not strong and a value closer to one means the method is strong (Shah and Pathak, 2010). The optimized composition (THQ-CPLNPs) prepared with PCL (A; 50 mg), CS (B; 0.5%), and PVA (C; 2.5%) exhibited the particle size of 182.32 ± 6.46 nm, PDI of 0.179 ± 0.012, and EE of 79.86% ± 4.36%, respectively. The software examined an analysis of variance (ANOVA) for all three responses (Y_1_, Y_2_, and Y_3_), and the obtained data indicated that the quadratic model was well fitted (Table 4). Figure 2 represents the quantitative comparison of the responses of the experimental values with the predicted values.

**Figure 3.**
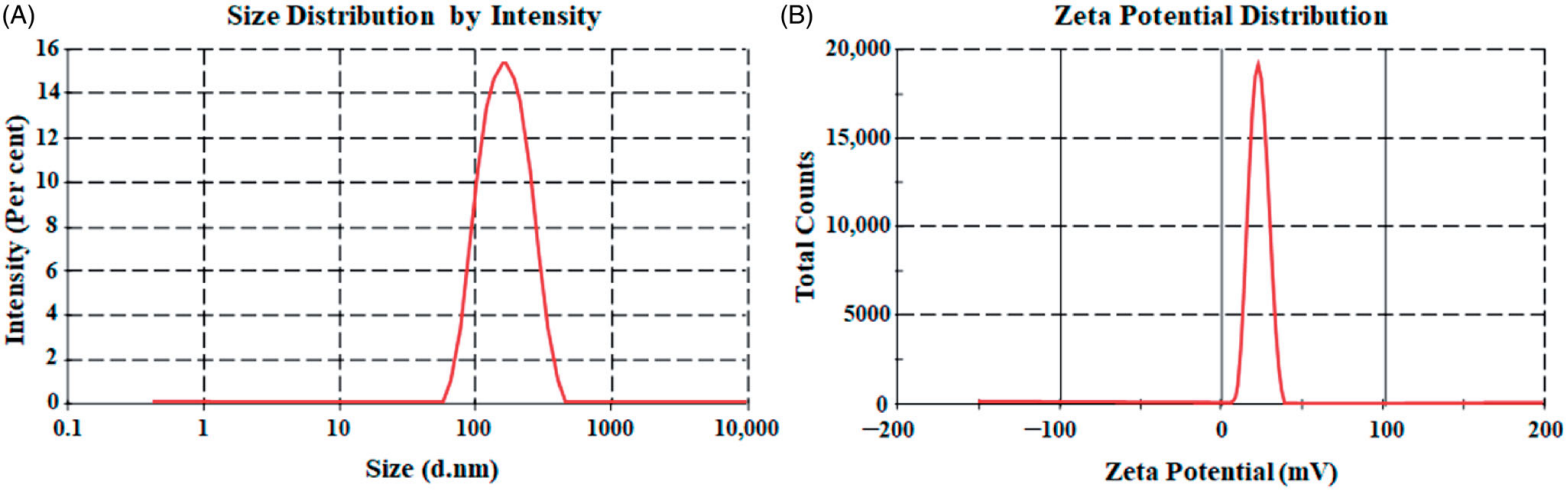
Particle size (A). Zeta potential (B) of optimized thymoquinone chitosan-polycaprolactone nanoparticles (THQ-CPLNPs)..

### 3.2. Characterization

#### 3.2.1. Particle evaluation

The size and PDI of prepared THQ‐CPLNPs were found in the range of 144.56‒227.15 nm, and 0.139‒0.228, respectively (Table 2). The optimized THQ‐CPLNPs showed PS and PDI of 182.32 ± 6.46 nm (Figure 3A), and 0.179 ± 0.012, respectively. The small size and polydispersibility index (PDI) of NPs are two of the essential parameters for effective oral delivery of phytochemicals. The small PS indicates a higher surface area for absorption from the small intestine and low PDI indicates the homogeneous distribution of NPs. The polymer-based NPs system with PDI value <0.3 represents the good uniformity between the particles (Danaei et al., 2018). Moreover, optimized THQ‐CPLNPs showed a zeta potential (ZP) of +21.36 ± 1.22 mV (Figure 3B). A positive ZP on THQ-CPLNPs was due to the presence of chitosan which is a cationic natural polymer. A positive charge increases the adhesion of NPs to the intestinal mucosa which in turn provides significantly higher residence time to absorb the drug from the intestine (Lima et al., 2018). The above finding is following a previously published investigation (Alshehri et al., 2020). They have reported improved delivery of thymoquinone by preparing chitosan coated NPs. The zeta potential of optimized formulation was observed to be +12.24 ± 2.32 mV.

#### 3.2.2. Encapsulation and loading efficiency

EE of the developed THQ-CPLNPs was observed in the range of 70.47‒92.37% (Table 2). The optimized THQ-CPLNPs showed the EE and DL value of 79.86% ± 4.36% and 13.45 ± 1.38%. The higher EE is due to the entrapment of THQ in to the polymer matrix. The coating with chitosan forms a layer around the NPs and inhibits the diffusion of THQ from CPLNPs (Delan et al., 2020).

#### 3.2.3. Drug release

**Figure 4.**
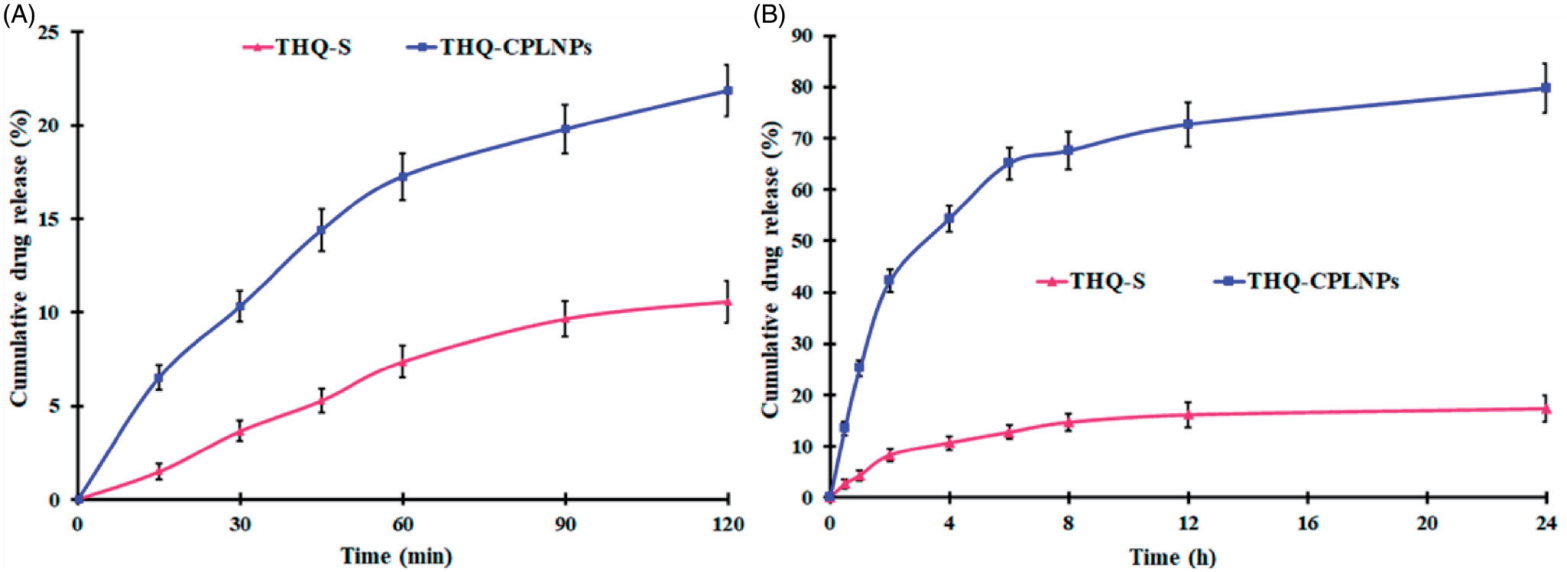
Thymoquinone suspension (THQ-S) and optimized thymoquinone chitosan-polycaprolactone nanoparticles (THQ-CPLNPs). (A) 0.1 N HCl, (B) Phosphate buffer saline.

The release behavior of THQ from optimized THQ-CPLNPs in SGF and SIF is depicted in Figure 4. In SIF (pH 6.8), THQ-CPLNPs showed a biphasic pattern, with an initial burst (42.226 ± 2.17 in 2 h), followed by a sustained release up to 24 h, reaching a maximum oil release of 79.65 ± 4.72%. The sustained release after 2 h to 24 h was due to the THQ encapsulated in the inner core of the polymeric matrix, which was released slowly by slow diffusion. Also, the slow release of THQ from THQ-CPLNPs was possible because of the CS coating that protects the THQ from desorption and diffusion from the NPs system (Alshehri et al., 2020). In SIF (pH 1.2), THQ-CPLNPs showed only 21.84 ± 1.37% release after 120 min. CS protects the THQ in the SGF in the NPs and inhibits significant release. Interaction between CS which is used to coat the NPs interacts with lipids present in the stomach that results in the limited release of THQ from the NPs (de Souza et al., 2020). On the other hand, THQ release from THQ-S was found to be less than 20% both in SGF as well as in SIF. This can be explained by the fact that THQ is a water-insoluble drug and its dissolution is limited due to poor solubility (Fakhria et al., 2019). The correlation coefficient (*R*^2^) of various mathematical kinetic models are compiled in Table 5. As per the obtained results, the Korsmeyer–Peppas model represents the highest R^2^ (0.9686) and was considered as the best-fitted model. Moreover, the calculated exponents “n” obtained from the Korsmeyer–Peppas kinetic model was found 0.293, suggesting that the mechanism of THQ release from CPLNPs was Fickian diffusion.

**Table 5.** Release kinetic models fitting in terms of linear regression coefficient (R^2^).

		Plot		
Model	Equation	X-axis	Y-axis	R^2^
Zero order	$M_{t}=M_{0}+k_{0}\;t$	Fraction of drug released	time	0.6335
First order	$\ln\;M_{t}=\ln\;M_{0}+\;k_{1}\;t$	Log % drug remaining	time	0.7965
Korsmeyer–Peppas model	$M_{t}/M_{\infty }={\mathrm{kt}}^{n}$	Log fraction of drug released	log time	0.9686
Higuchi matrix model	$M_{t}=M_{0}+k_{H}t^{1/2}$	Fraction of drug released	$\sqrt{t\mathrm{ime}}$	0.8379
Best fitted model		Korsmeyer–Peppas model		

#### 3.2.5. Mucoadhesion study

The mucoadhesive behavior of THQ-CPLNPs and THQ-S was studied through mucus glycoprotein assay. As expected, THQ-CPLNPs revealed a significantly higher (*p* < 0.05) mucoadhesive efficiency (79.27 ± 3.52%) compared to THQ-S (14.03 ± 1.21%). The coating of PL-NPs with chitosan provided a significantly higher binding efficiency to mucin. The reason for high mucoadhesion showed by THQ-CPLNPs due to the electrostatic interaction between cationic chitosan with the anionic mucin. The amino group of chitosan adsorbed to the sialic acid of mucin and help to get greater mucoadhesion (Pauluk et al., 2019). Moreover, the hydrophilic nature of CS further strengthens the hydrogen bond between THQ-CPLNPs and mucin molecules hence improves mucoadhesion (Sudhakar et al., 2020). The higher mucoadhesive nature helps to get a longer residence time in the gastrointestinal tract (GIT) that leads to higher absorption of THQ, therefore, it will help to enhance the bioavailability of THQ. Our results showed similar findings to previously reported research literature (Yu et al., 2017; Pauluk et al., 2019). They have reported the enhanced mucoadhesion of chitosan-coated resveratrol and cetirizine NPs than uncoated NPs. The enhanced mucoadhesive property helps to improve drug absorption and bioavailability. The chitosan-coated NPs can interact with the mucus and retain at the site of action lead to enhanced absorption and bioavailability (Pauluk et al., 2019).

#### 3.2.6. Permeation study

**Figure 5.**
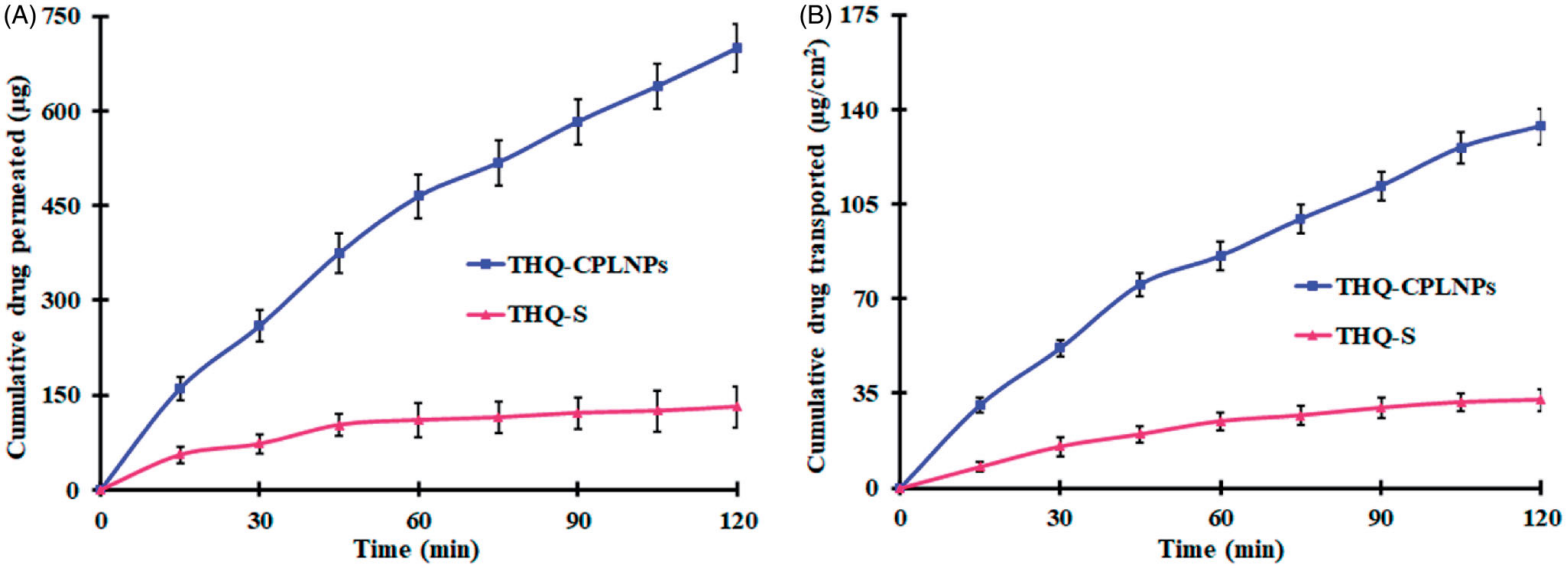
Thymoquinone suspension (THQ-S) and optimized thymoquinone chitosan-polycaprolactone nanoparticles (THQ-CPLNPs). (A) THQ permeated, (B) THQ transported.

The intestinal permeability of THQ-CPLNPs and THQ-S was performed on the rat intestine, and the THQ permeability profiles are depicted in Figure 5(A,B). The permeated amount of THQ from THQ-CPLNPs was found to be 698.62 ± 37.87 μg, which is 5.32 fold higher (*p* < .05) than THQ-S (131.28 ± 32.57 μg). Similarly, THQ-CPLNPs exhibited 133.65 ± 6.63 µg/cm^2^ which is 4.11-fold higher permeation than neat THQ suspension (32.48 ± 4.16). Also, THQ-CPLNPs revealed 4.21-fold enhanced $P_{\mathrm{app}}$ compared to THQ-S. Higher intestinal permeation with THQ-CPLNPs was achieved due to nanosized particles which provided a higher surface area that results in significantly higher absorption of THQ. THQ entrapped in chitosan NPs, it is not available for P-gp pumps and easily transported across the intestinal wall (Zare et al., 2018). Also, the mucoadhesive property of THQ-CPLNPs is responsible for the disruption and/or modulation of tightness in the tight junctions of the GI mucosa. The positive charge due to coating with CS, interact with negatively charged GI mucosa that leads to the opening of tight junctions (Gilani et al., 2021). Thus, THQ-CPLNPs revealed significantly higher THQ permeation from GI mucosa.

#### 3.2.7. Permeation depth

**Figure 6.**
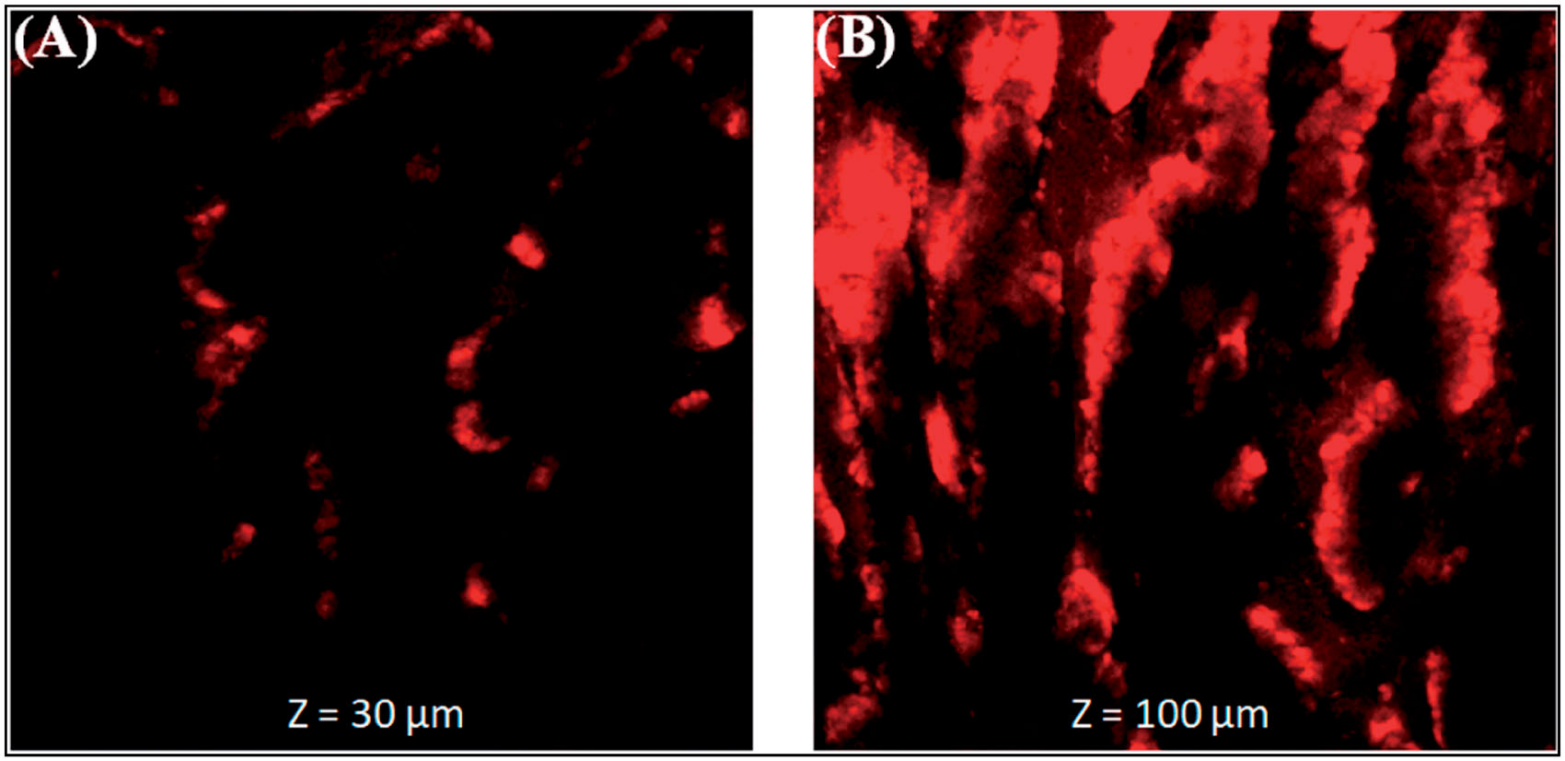
Permeation depth image (A). Thymoquinone suspension (THQ-S) (B). Optimized thymoquinone chitosan-polycaprolactone nanoparticles (THQ-CPLNPs).

Confocal microscopy of Rh-B loaded CPLNPs and Rh-B solution treated sliced section was conducted to verify the permeation of THQ, and the results were depicted in Figure 6(A,B). Rh-B loaded CPLNPs revealed much higher penetration (*z* = 100 μm) compared to free Rh-B solution (*z* = 30 μm). The higher penetration of CPLNPs was due to the nanometric sized particles that provided a higher surface area that results in significantly higher penetration into the intestine. Furthermore, the positive charge due to coating with CS, NPs interact with negatively charged GI mucosa that helps to the opening of tight junctions that results in higher intestinal penetration (Fakhria et al., 2019).

#### 3.2.8. Irritation study

**Figure 7.**
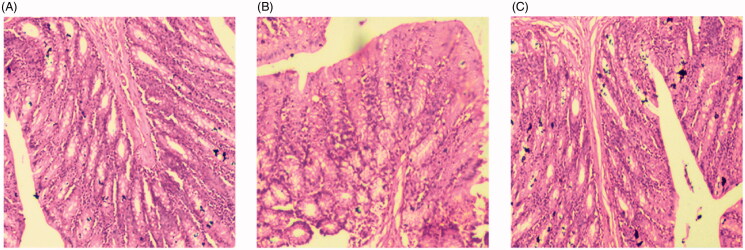
Gastric mucosa irritation study image of (A). Normal saline (B). Thymoquinone suspension (THQ-S) (C). Optimized thymoquinone chitosan-polycaprolactone nanoparticles (THQ-CPLNPs).

The gastric mucosa irritation study was performed on Wistar rats using normal saline, THQ-S and THQ-CPLNPs. The effects of the formulations were evaluated by comparing the histopathology of the gastric mucosa (Figure 7(A–C)). The image revealed nonsignificant changes in the mucosal lining, as well as no hemorrhagic erosion was observed. There was an intact structure of gastric mucosa was observed in all the treated group, and results were comparable to the normal saline-treated group. The good mucosal structural integrity revealed good biocompatibility of THQ-CPLNPs and THQ-S to the gastric mucosa of Wistar rats. Our findings are as per a previously published investigation (Sharma et al., 2019) .

#### 3.2.9. Pharmacokinetic study

**Figure 8.**
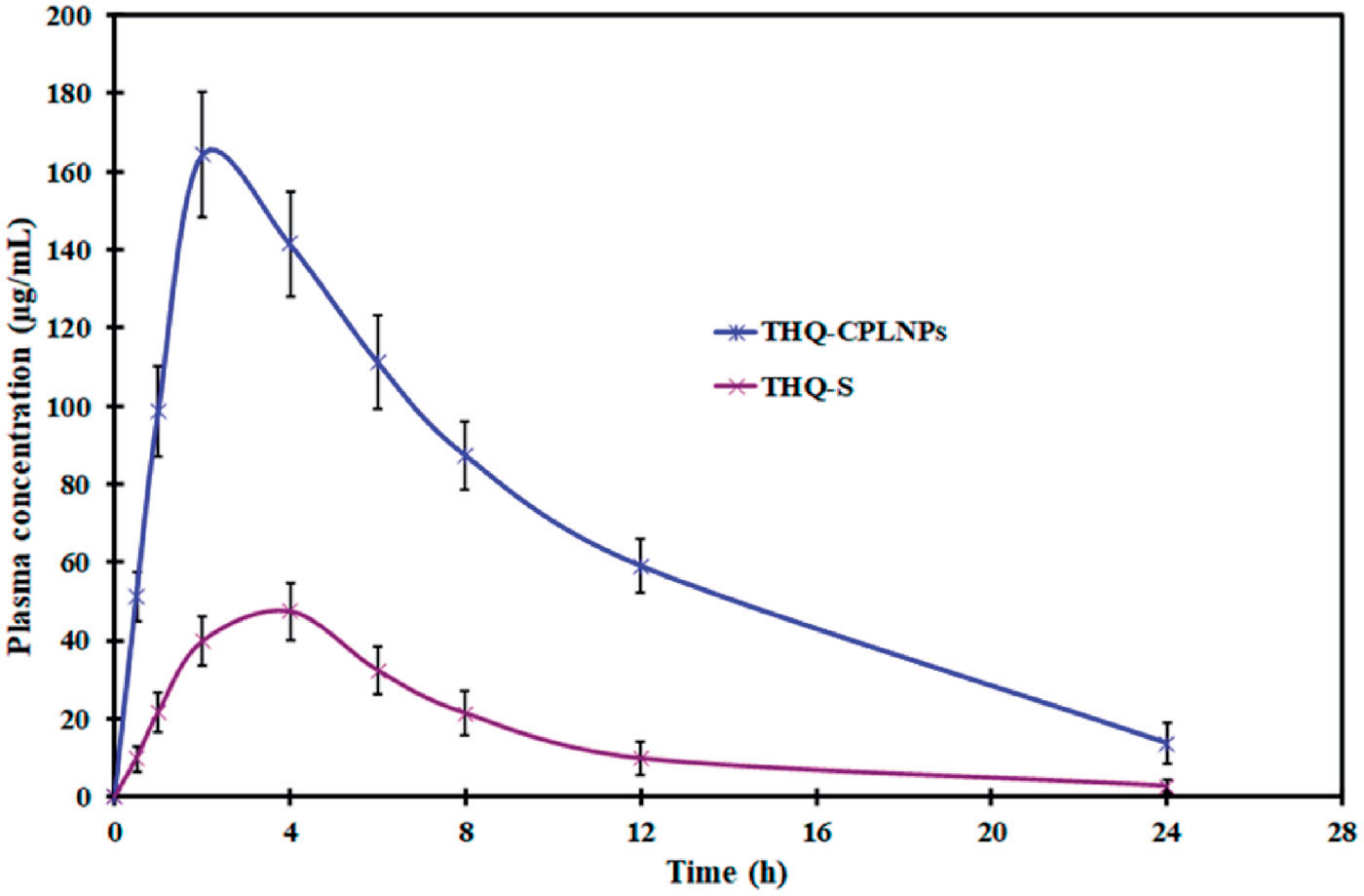
Comparative pharmacokinetic study of optimized thymoquinone chitosan-polycaprolactone nanoparticles (THQ-CPLNPs) and thymoquinone suspension (THQ-S).

The pharmacokinetic profiles of THQ following oral administration of THQ-S and THQ-CPLNPs are depicted in Figure 8, and parameters are represented in Table 6. A significant difference (*p* < .05) between the pharmacokinetic parameters of THQ-S and THQ-CPLNPs was observed. After a single dose oral administration, THQ-CPLNPs exhibited ∼3.4-fold higher (164.34 ± 8.74 µg/mL) compared to THQ-S (47.53 ± 4.27 µg/mL). Similarly, THQ-CPLNPs showed 4.17 fold higher AUC with the value of 1670.27 ± 71.95 µ.h/mL compared to THQ-S (400.34 ± 17.38 µ.h/mL), MRT, *t*_1/2_, and *K*_el_ after oral administration of neat THQ suspension were found to be 4 ± 0.00 h, 6.91 ± 0.73 h, 7.57 ± 0.67 h, and 0.091 ± 0.012 h^‒1^, respectively. While, MRT, t_1/2,_ and K_el_ for THQ-CPLNPs was observed to be 2 ± 0.00, 7.66 ± 0.57, 8.41 ± 0.85, and 0.082 ± 0.01 h^‒1^, respectively. The above results suggested that the oral administration THQ-CPLNPs resulted in remarkable improvement (*p* < .05) in the bioavailability of THQ when compared to THQ-S. Enhanced THQ bioavailability after oral administration THQ-CPLNPs was due to encapsulation of THQ into the small-sized particles that increase the solubility of THQ as well as the surface area that results in significantly higher absorption of THQ from GIT upon oral administration. Moreover, the mucoadhesive property of THQ-CPLNPs helps to increase residence time in GIT that leads to higher absorption, thereafter higher oral bioavailability of THQ (Murthy et al., 2020).

**Table 6.** Pharmacokinetic parameters of thymoquinone encapsulated chitosan modified polycaprolactone nanoparticles (THQ-CPLNPs) and THQ suspension (THQ-S).

Parameters	THQ-S	THQ-CPLNPs
$C_{\max}$ (µg/mL)	47.53 ± 4.27	164.34 ± 8.74*
$T_{\max}$ (h)	4 ± 0.00	2 ± 0.00*
${\mathrm{AUC}}_{0\to 24}$ (µ.h/mL)	400.34 ± 17.38	1670.27 ± 71.95*
${\mathrm{AUC}}_{0\to \infty }$ (µ.h/mL)	430.72 ± 19.54	1836.99 ± 82.17*
${\mathrm{AUMC}}_{0\to 24}$ (µ.h^2^/mL)	2766.22 ± 172.78	12807.05 ± 373.68*
${\mathrm{AUMC}}_{0\to \infty }$ (µ.h^2^/mL)	3827.38 ± 211.32	18831.29 ± 423.53^a^
MRT (h)	6.91 ± 0.73	7.66 ± 0.57
$t_{1/2}$ (h)	7.57 ± 0.67	8.41 ± 0.85
$K_{\mathrm{el}}$ (h^‒1^)	0.091 ± 0.012	0.082 ± 0.01

^a^Denotes highly significant (*p* < .05) values of optimized TQ-CPLNPs when compared with THQ-S.

## 4. Conclusion

In this study, THQ-CPLNPs was successfully developed and optimized by 3^3^-BBD. The optimized THQ-CPLNPs has shown desirable size, PDI, surface charge, and encapsulation efficiency. THQ showed a biphasic release pattern of THQ from CPLNPs, with initial burst release followed by prolonged release up to 24 h. Chitosan represents a significant role in the enhancement of mucin adsorption on NPs surface and THQ-CPLNPs showed an excellent mucoadhesion profile. THQ-CPLNPs showed significantly enhanced intestinal permeation and this result was further confirmed by confocal microscopy. Gastric irritation study confirmed the safety of THQ-CPLNPs as well as showed 3.5 fold higher relative oral bioavailability compared to THQ-S. Based on our findings, it is suggested that chitosan-modified PLNPs could be an excellent nanoplatform to improve the oral bioavailability of THQ.
